# Shared decision-making in palliative cancer care: A systematic review and metasynthesis

**DOI:** 10.1177/02692163241238384

**Published:** 2024-03-13

**Authors:** Jannicke Rabben, Bella Vivat, Mariann Fossum, Gudrun Elin Rohde

**Affiliations:** 1 Department of Health and Nursing Science, Faculty of Health and Sport Sciences, University of Agder, Kristiansand/Grimstad, Vest-Agder, Norway; 2 Marie Curie Palliative Care Research Department, Division of Psychiatry, University College London, London, UK; 3 Department of Clinical Research, Sorlandet hospital, Kristiansand, Vest-Agder, Norway

**Keywords:** Decision making, neoplasms, palliative care, qualitative research, shared decision making, systematic review

## Abstract

**Background:**

Shared decision-making is a key element of person-centred care and promoted as the favoured model in preference-sensitive decision-making. Limitations to implementation have been observed, and barriers and limitations, both generally and in the palliative setting, have been highlighted. More knowledge about the process of shared decision-making in palliative cancer care would assist in addressing these limitations.

**Aim:**

To identify and synthesise qualitative data on how people with cancer, informal carers and healthcare professionals experience and perceive shared decision-making in palliative cancer care.

**Design:**

A systematic review and metasynthesis of qualitative studies. We analysed data using inductive thematic analysis.

**Data sources:**

We searched five electronic databases (MEDLINE, EMBASE, PsycINFO, CINAHL and Scopus) from inception until June 2023, supplemented by backward searches.

**Results:**

We identified and included 23 studies, reported in 26 papers. Our analysis produced four analytical themes; (1) Overwhelming situation of ‘no choice’, (2) Processes vary depending on the timings and nature of the decisions involved, (3) Patient-physician dyad is central to decision-making, with surrounding support and (4) Level of involvement depends on interactions between individuals and systems.

**Conclusion:**

Shared decision-making in palliative cancer care is a complex process of many decisions in a challenging, multifaceted and evolving situation where equipoise and choice are limited. Implications for practice: Implementing shared decision-making in clinical practice requires (1) clarifying conceptual confusion, (2) including members of the interprofessional team in the shared decision-making process and (3) adapting the approach to the ambiguous, existential situations which arise in palliative cancer care.

**What is already known about the topic?**

- Shared decision-making is generally promoted as a favoured model for preference-sensitive decisions across many health care settings internationally and is described as a key element of person-centred care.
- Individual and systemic barriers may limit the implementation of shared decision-making in practice, both generally, and in palliative cancer care in particular.

**What this paper adds?**

- Decision-making in palliative cancer care is characterised by limited equipoise and awareness of choice, and shared decision-making in this context is a complex process involving multiple decisions over time.
- The complexity of decision-making in palliative cancer care requires adapting the approach to shared decision-making to variable and individual situations.
- The person with palliative cancer and their physician are central to treatment decision-making, but other healthcare professionals, like nurses, have an important role in facilitating and supporting shared decision-making when deciding about treatment and care.

**Implications for practice, theory or policy**

- Palliative cancer care professionals should pay more attention to creating awareness of choice and supporting involvement of people with cancer and their families in decision-making.
- Trusting relationships between people with palliative cancer and health professionals, including strong communication skills, are needed to engage people with palliative cancer and their families in shared decision-making.
- The interprofessional team of physicians, nurses and other health professionals need a clear understanding of what shared decision-making is, and the involvement of all team members in the process must be complementary.

## Background

Shared decision-making is generally promoted as a favoured model for preference-sensitive decisions across many health care settings internationally,^1^ and it has been described as a key element of person-centred care.^2,3^ In previous research mostly conducted in Western/Northern health care settings, shared decision-making is commonly defined as an approach where clinicians and patients make decisions together using the best available evidence.^4^ A previous review of shared decision-making models and definitions identified several central components, including: creating awareness of choice, describing treatment options, learning about the person receiving care, tailoring information, discussing the preferences of people receiving care, deliberation and making or deferring the decision.^5^

Several actions have been proposed for increasing the involvement of people receiving care in shared decision-making, for example, development of decision aids, and skills training of healthcare professionals.^1^ However, the effectiveness of interventions to increase shared decision-making is uncertain, and patient involvement still limited.^1,6^ Healthcare professionals can express positive atti-tudes toward shared decision-making, but report difficulties with practising shared decision-making in clinical work.^7,8^ A wide range of individual, organisational and system levels barriers to implement shared decision-making in routine practice have been identified. For clinicians, these include: limited skills, knowledge or formal training and limited resources, including time.^9,10^ Patients may have limited health literacy, awareness of choice or belief in their capacity to be involved in decision-making.^9,10^

Previous reviews have shown that most people receiving care prefer an active or shared role in health-related decision-making, but reported preferences vary by patient population, study design and the preference measure used.^11–13^ The preferred roles of people with cancer in decision-making may also vary by country and ethnic or cultural background.^14,15^ People with cancer may also experience a lack of congruence between their preferred and perceived level of involvement.^12,16^

Shared decision-making in palliative care is even more challenging for everyone involved, people with palliative cancer, their families and clinicians. This is for a range of reasons. The decisions required may be multiple and complex, related to treatment options, treatment breaks or discontinuation, clinical trial participation, symptom management or place of care.^17–19^ A common approach in cancer care is to use systemic anticancer treatment with palliative intent.^20^ However, studies show limitations in communicating with and involving people receiving palliative cancer treatment, who may sometimes believe the treatment has a curative intent or overestimate its potential benefit.^21,22^ Profound uncertainty and serious illness can also reduce decisional capacity, hindering involvement and limiting truly shared decision-making.^23,24^

Previous systematic reviews focussing specifically on shared decision-making in palliative care or oncology have explored literature addressing barriers and facilitators,^25^ preferred and perceived level of involvement of people with cancer,^13,18,26^ specific decisions like withdrawal of therapy or clinical trial participation,^19,27^ or, most recently, the use of decision aids.^28^ In their review, for palliative care in general, Kuosmanen et al.^29^ included the perspectives of all three relevant groups: people receiving palliative care, their families and healthcare professionals.

Our review focusses specifically on qualitative research relevant to the research question: How do patients, informal carers and healthcare professionals experience and perceive shared decision-making in palliative cancer care? That is, not all palliative care, but specifically cancer care. We aimed to integrate the perspectives of all relevant stakeholders, because shared decision-making emerges from the interactions between all those involved. Our review focussed particularly on cancer treatment and care, in order to understand how shared decision-making is experienced and perceived in this specific context. This narrow scope enabled an integration of qualitative findings and the creation of deeper knowledge, strengthening the metasynthesis.

Note: we use the term ‘palliative cancer’ throughout this paper to signify advanced, non-curative cancer.

## Methods

We conducted a metasynthesis, guided by the Sandelowski and Barroso framework.^30^ A metasynthesis enables an interpretive integration of qualitative findings resulting in novel interpretations.^30^ Following this framework, we applied the following steps: (1) systematically searching for and retrieving relevant reports of qualitative studies, (2) individually and comparatively appraising papers, (3) classifying the findings and (4) extracting and synthesising findings.

We registered our study protocol in the International Prospective Register of Systematic Reviews (PROSPERO), registration number CRD42022321483.^31^ We follow the Enhancing Transparency in Reporting the Synthesis of Qualitative Research (ENTREQ) Statement in reporting this review.^32^

### Data sources and search strategy

We conducted an initial comprehensive bibliographic search of the databases MEDLINE, EMBASE, PsycINFO (Ovid), CINAHL (EBSCOhost) and Scopus from inception to August 3rd 2022. After identifying and selecting relevant keywords, we designed strategies to suit each database in collaboration with a librarian. (A table of keywords and detailed search strategy for each database is available in Supplemental File A). We re-ran the search on June 1st, 2023, to check for any new publications in the interim.

### Inclusion and exclusion criteria

We included papers if they (1) were peer reviewed scientific papers reporting qualitative or mixed-methods empirical studies; (2) reported on study populations which were either adults with palliative cancer, informal carers (including bereaved) of people with palliative cancer or health care professionals working with people with palliative cancer in any health care setting; (3) included qualitative findings about decision-making involving both patients (and informal carers) and health care professionals. We excluded (1) study designs: reviews, quantitative studies, surveys, studies with observational data only and patient case studies; (2) studies of interprofessional decision-making or surrogate decision-making, advance directives/advance care planning and (3) papers written in any language other than English or any Scandinavian language.

### Study selection

Two reviewers (JR and GR) screened title and abstract of identified records using the Rayyan tool.^33^ Full text papers were independently assessed for eligibility, then decisions reached collaboratively. The included studies did not all aim to describe experiences and perspectives of shared decision-making, nor did all describe how they defined shared decision-making. Methodologically, this led to reading more papers in full text and making judgements on what findings were related to shared decision-making, guided by previously described ‘key elements’.^5^ Disagreements were resolved by discussion until consensus was reached, involving a third research team member (MF). Reason for exclusion was stated for each record. We conducted backward searching of the included papers, and assessed and in-cluded any eligible additional papers found.

### Individual and comparative appraisal

Following the reading guide provided by Sandelowski and Barosso,^30^ each paper was individually appraised by two independent reviewers (either JR/GR or JR/MF). The reading guide provides a comprehensive list of appraisal parameters that can be systematically used to conduct a judicious appraisal of qualitative research papers.^30^ The mixed methods papers were appraised on the reporting of the qualitative element of their studies. We operationalised the reading guide into 13 Yes/No questions. Sandelowski and Barroso’s approach seeks not to appraise study quality *per se*, but to identify all potentially relevant findings, so, as part of the appraisal, we discussed and classified the findings according to Sandelowski and Barroso’s^34^ typology of findings. Reasons for exclusion during appraisal would be if the findings in the report were classified as ‘No findings’, ‘Topical survey’ or ‘Thematic survey’.^34^ The three reviewers discussed and reconciled any disagreements.

We then conducted a comparative appraisal of all included studies, organising and displaying key elements of information in each paper together. Extraction of study characteristics were performed by the first author (JR), and another team member (BV) checked sections of the extracted information for accuracy. The comparative appraisal provides an interpretive context for the synthesis.^30^

### Data extraction and analysis

We imported included papers into NVivo QDA software for extracting findings,^35^ and further analysis. We extracted both illustrative quotes and study authors’ interpretations of study participants’ experiences related to shared decision-making, including experiences of limitations to or lack of shared decision-making. Quotations are therefore either illustrative quotes from the original study, or quotes from study authors’ interpretive text; we indicate which for each quote. We linked extracted data to the relevant participant population: patients, informal carers or health care professionals, to enable exploration of the contribution of each population to the themes. Aided by the crosstab matrix function in NVivo, we created a visualisation of how the findings related to these three populations, independent of studies.

We analysed data thematically, using the analytical approach described by Thomas and Harden.^36^ This involves (1) an inductive line-by-line coding of findings and development of descriptive themes, grouping them into a hierarchical tree structure and (2) generating analytical themes.^36^ The first author (JR) performed the data extraction, coding and analysis. One member of the team (MF) independently coded parts of the data for comparison and discussion to validate the extraction and coding process. The whole research team then discussed the initial descriptive and analytical themes, thus increasing the trustworthiness of the analysis by exa-mining the first author's preliminary interpretations. Following the updated search, we coded findings from new included studies and integrated them into the existing codes and themes. This resulted in minor changes in codes, but no additional or revised themes.

## Results

### Study characteristics and metasummary

**Figure 1.**
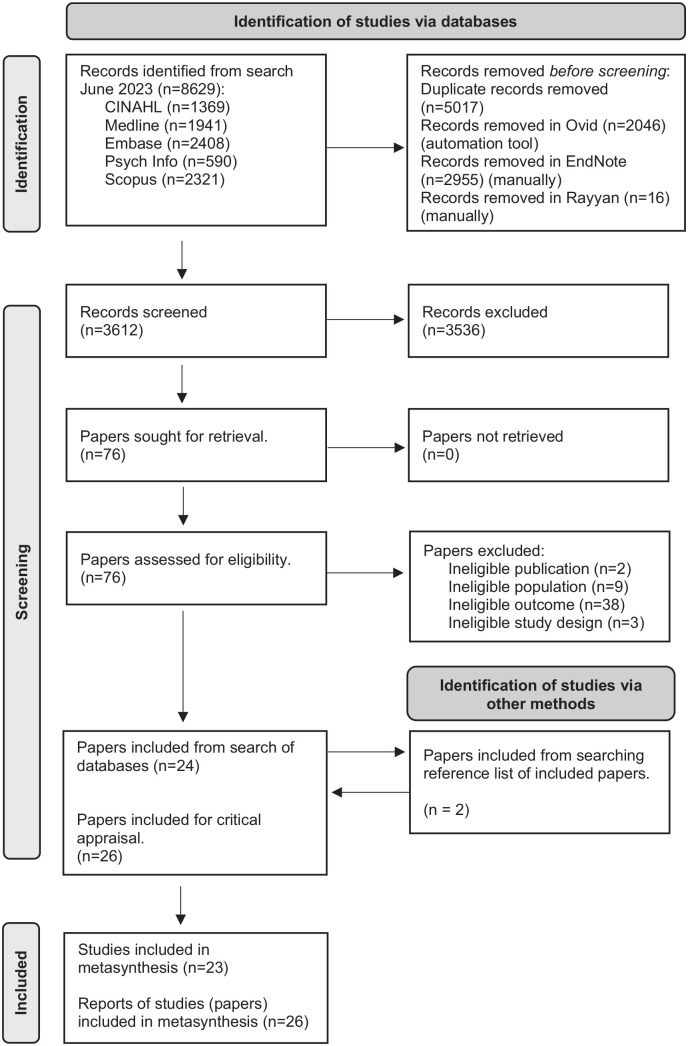
Screening and inclusion flow chart.

From 3612 records and 76 full text papers assessed for eligibility, we included a final set of 23 studies, reported in 26 papers. Figure 1 presents a Flow-chart showing screening and inclusion.

Table 1 summarises the characteristics of the 23 studies included in this review. Twenty studies were qualitative, and three mixed methods. All were published in the last 21 years; seven papers between 2003 and 2010, and 19 between 2015 and 2023. Studies were conducted in Europe (14 studies), North America (7 studies) or Australasia (2 studies). Fourteen studies focussed on treatment decision-making, and nine included decisions related to treatment and care, participation in clinical trials and/or symptom management and care. Participants were patients (*n* = 276), informal carers (*n* = 195) and healthcare professionals (*n* = 305).

**Table 1.** Characteristics of included papers.

Author (year)	Country^a^	Related to (same study)	Sample^b^					Design and methods	Decision^a^
			Patients	Informal carers	Physicians	Nurses	Other HCPs		
			*n*	*n*	*n*	*n*	*n*		
Al Achkar et al. (2022)	US		25					Qualitative, deductive and inductive coding	Treatment and care; second opinion
Barthow et al. (2009)	NZ	McCullough et al. (2010)				13^c^		Qualitative, content analysis	Treatment; variable
Beaussant et al. (2015)	France		29		17			Mixed-method, grounded theory for qualitative	Treatment; pursuit, limit or withholding
Boele et al. (2023)	UK		15	13	3	2		Qualitative, reflexive thematic analysis	Treatment; anti-tumour treatment
Bos-van den Hoek et al. (2021)	NL	Bos-van den Hoek et al. (2022)				18		Qualitative, thematic analysis	Treatment; variable
Bos-van den Hoek et al. (2022)	NL	Bos-van den Hoek et al. (2021)			15			Qualitative, thematic analysis	Treatment; variable
Brom et al. (2017)	NL	De Snoo-Trimp et al. (2015)	14		18^c^			Qualitative, Open coding analysis	Treatment; 2^nd^ and 3rd line chemotherapy
Chen et al. (2021)	US		17					Qualitative, thematic analysis	Treatment; palliative radiotherapy
De Kort et al. (2010)	NL		13		19	8	3	Qualitative, constant comparative analysis	Treatment; course of treatment
De Snoo-Trimp et al. (2015)	NL	Brom et al. (2017)			12^c^	1	1	Qualitative, Open coding analysis	Treatment; chemotherapy
Elit et al. (2003)	Canada		21					Qualitative, content analysis	Treatment; advanced ovarian cancer
Elit et al. (2010)	Canada		26					Qualitative, content analysis	Treatment; recurrent ovarian cancer
Gregersen et al. (2022)	Denmark		9					Qualitative, thematic analysis	Clinical trial participation
Haun et al. (2022)	Germany			16				Mixed-method, content/thematic analysis	Treatment; aggressive treatment at EOL
Kvåle & Bondevik, (2008)	Norway		20^d^					Qualitative (Phenomenological) Giorgi’s scientific approach	Treatment and care; interventions/management
Lape et al. (2020)	US		23					Qualitative, thematic analysis	Treatment; spinal metastases
LeBlanc et al. (2018)	US		17	14	10			Mixed-method, content analysis for qualitative data	Treatment; variable
Lee et al. (2009)	Australia		14	7	4	12	2	Qualitative (Grounded theory), constant comparative analysis	Treatment and care; palliative care
Løwe et al. (2021)	Denmark		13		15	15		Qualitative (Phenomenological-hermeneutic), Ricoeur’s theory of interpretation	Treatment; advanced prostate cancer
McCullough et al. (2010)	NZ	Barthow et al. (2009)			8	13^c^		Qualitative, content/thematic analysis	Treatment; variable
Norton et al. (2019)	US			92				Qualitative, three-phased coding approach	Treatment and care; stopping/EOL-transition
Pfeil et al. (2015)	Germany				12	6		Qualitative (Grounded theory), constant comparative analysis	Treatment and care; EOL-decisions
Robijn et al. (2018)	Belgium, UK, NL			24	26	30		Qualitative, content analysis	Symptom-management and care; continuous sedation
Sowerbutts et al. (2020)	UK		20	13	7	14	11	Qualitative, thematic analysis (VanManen)	Symptom-management and care; parental nutrition
Tarberg et al. (2022)	Norway				13			Qualitative (Hermeneutic), thematic analysis	Treatment and care; variable decisions in care pathway
Van Oosterhout et al. (2021)	NL			16				Qualitative, constant comparative analysis	Treatment; variable
			276	195	169^e^	119^e^	17		
Total HCPs ^e^					305				
Total participants ^e^			776						

a US: United States, NZ: New Zealand; UK: United Kingdom; NL: Netherlands; HCPs: Healthcare professionals; EOL: end-of-life.

b Terms: physician is used in a broad sense, and include oncologists, surgeons and other medical practitioners like general practitioners. Nurses include registered nurses, specialist nurses and nurse practitioners. Informal carers include people covered by all terms used in included papers; relatives, family members/ caregivers and bereaved. Other HCPs include radiotherapists, counsellors, dieticians and managers.

c Overlapping sample of thirteen nurses in Barthow et al. (2009) and McCullough et al. (2010) and ten physicians interviewed in De Snoo-Trimp et al. (2015) and Brom et al. (2017).

d Of these, sixteen were palliative.

e Adjusted for overlapping samples.

Table 2 details the critical appraisal of the 20 qualitative studies and the qualitative aspects of the three mixed methods studies. No papers were excluded following appraisal or classifying findings. More details on included studies’ aims, methodology, theoretical frameworks and study settings, are provided in Supplemental File B.

**Table 2.** Critical appraisal of included studies.

Author, year	Problem, purpose^a^	Theoretical orientation	Methodological orientation	Sampling strategy, sample	Data collection	Analysis	Techniques for validation	Findings	Discussion and Implication	Ethics	Type of findings^b^
Al Achkar et al. (2022)	+^c^	+	+	+	+	+	+	+	+	+	TD
Barthow et al. (2009)	+	−^c^	+	+	+	+	+	+	+	+	CD
Beaussant et al. (2015)	+	−	+	+	+	+	+	+	+	+	TD
Boele et al. (2023)	+	−	+	+	+	+	+	+	+	+	TD
Bos-van den Hoek et al. (2021)	+	−	+	+	+	+	+	+	+	+	TD
Bos-van den Hoek et al. (2022)	+	−	+	+	+	+	+	+	+	+	TD
Brom et al. (2017)	+	+	+	+	+	+	+	+	+	+	TD
Chen et al. (2021)	+	−	+	+	+	+	+	+	+	+	TD
De Kort et al. (2021)	+	−	+	+	+	+	+	+	+	+	TD
De Snoo-Trimp et al. (2015)	+	+	+	+	+	+	+	+	+	+	TD
Elit et al. (2003)	+	−	+	+	+	+	+	+	+	+	TD
Elit et al. (2010)	+	−	+	+	+	+	+	+	−	+	TD
Gregersen et al. (2022)	+	−	+	+	+	+	+	+	+	+	IE
Haun et al. (2022)	+	+	+	+	+	+	+	+	+	+	IE
Kvåle & Bondevik (2008)	+	−	+	+	+	+	+	+	+	+	TD
Lape et al. (2020)	+	−	+	+	+	+	+	+	+	+	TD
LeBlanc et al. (2018)	+	−	-	+	+	+	−	+	+	+	TD
Lee et al. (2009)	+	−	+	+	+	+	+	+	+	+	IE
Løwe et al. (2021)	+	+	+	+	+	+	−	+	+	+	TD
McCullough et al. (2010)	+	−	+	+	+	+	+	+	+	+	CD
Norton et al. (2019)	+	−	+	+	+	+	−	+	+	+	TD
Pfeil et al. (2015)	+	−	+	+	+	+	−	+	+	+	TD
Robijn et al. (2018)	+	+	+	+	+	+	+	+	+	+	CD
Sowerbutts et al. (2020)	+	−	+	+	+	+	+	+	+	+	TD
Tarberg et al. (2022)	+	+	+	+	+	+	+	+	+	+	IE
Van Oosterhout et al. (2021)	+	+	+	+	+	+	+	+	+	+	IE

a First three appraisal questions grouped in a single column.

b TD: thematic description; CD: conceptual description; IE: interpretive explanation.

c +: addressed; −: not addressed/missing.

### Findings from thematic metasynthesis

Our thematic metasynthesis concluded that shared decision-making with people who have advanced, non-curative cancer is a complex process of many decisions in a challenging, multifaceted and evolving situation where equipoise and choice are limited. The analysis produced four analytical themes: (1) Overwhelming situation of ‘no choice’, (2) Processes vary depending on the timings and nature of the decisions involved, (3) Patient-physician dyad is central to decision-making, with surrounding support and (4) Level of involvement depends on interactions between individuals and systems. Table 3 details the codes, and descriptive and analytical themes and Table 4 indicates which study findings contributed to which theme.

**Table 3.** Codes and descriptive and analytical themes.

Examples of codes	Descriptive themes	Analytical themes
Feeling a sense of urgency, time pressure and fear of decisional regret Feeling overwhelmed Strong emotions of fear, anxiety and grief	Overwhelming, emotional and stressful situation	1. Overwhelming situation of ‘no choice’
A No choice-situation Limited discussions of pros and cons Providing treatment, and not addressing uncertainty and other options to protect patients hope	Challenge of creating awareness of choice in a ‘No choice’ situation	
Patients want treatment Following treatment protocols, stopping treatment is failure Weighing quality of life over limited potential benefit	Mutual wish to treat and ‘fight against cancer’	
Many decisions where options may change Preferred and actual involvement is variable over time Revisiting information as the situation changes	A process over time	2. Processes vary depending on the timings and nature of the decisions involved
SDM in When and How, and everyday care decisions Less benefit, more open to patient preference Preferred and actual involvement is variable over type of decision	Variation depending on issue requiring decision	
Preferring and trusting the oncologist to decide Believe to know the patient’s preference for treatment Being involved at preferred level Perceived lack of skills or knowledge	The oncologist is the trusted expert and responsible for decision-making.	3. Patient-physician dyad is central to decision-making, with surrounding support
Variable involvement and support from family, social network and other patients Motivating, promoting and creating awareness of choice Clarify, deliberate and check quality of decision in formal and informal talks Provide information to the physician and advocate for patients’ perspective	Facilitation and support by other healthcare professionals/network	
Trusting relationship and continuity Being kind and able to share emotional involvement Communication skills and person-centred communication Experiential knowledge	The importance of personal competencies and a trusting relationship	4. Level of involvement depends on interactions between individuals and systems
Influenced by organisation and structure Nurses’ role is influenced by physicians’ attitudes and behaviour Teamwork and a supportive culture towards involvement Need for shared understanding of treatment goals Staff time is an essential precondition	Organisation and culture influence possibilities and roles in SDM	

**Table 4.** Studies’ contribution to analytical themes.

Analytical themes Author, year	1. Overwhelming situation of ‘no choice’	2. Processes vary depending on the timings and nature of the decisions involved	3. Patient-physician dyad is central to decision-making, with (. . .)	4. Level of involvement depends on interactions between (. . .)
Al Achkar et al. (2022)	x		x	x
Barthow et al. (2009) and McCullough et al. (2010)	x	x	x	x
Beaussant et al. (2015)	x		x	x
Boele et al. (2023)	x	x	x	x
Bos-van den Hoek et al. (2021, 2022)	x	x	x	x
Chen et al. (2021)			x	x
De Kort et al. (2010)	x	x		
Elit et al. (2003)	x	x	x	
Elit et al. (2010)	x	x	x	x
Gregersen et al. (2022)	x		x	x
Haun et al. (2022)	x		x	
Kvåle and Bondevik, (2008)		x	x	
Lape et al. (2020)	x		x	
LeBlanc et al. (2018)	x		x	
Lee et al. (2009)				x
Løwe et al. (2021)	x	x	x	x
Norton et al. (2019)	x	x	x	x
Pfeil et al. (2015)	x	x	x	x
Robijn et al. (2018)	x	x	x	
De Snoo-Trimp et al. (2015) and Brom et al. (2017)	x	x	x	
Sowerbutts et al. (2020)	x		x	
Tarberg et al. (2022)	x	x	x	x
Van Oosterhout et al. (2021)	x		x	x

**Figure 2.**
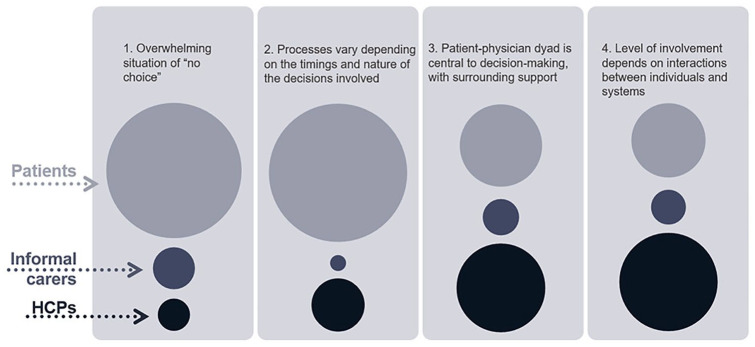
Visualisation of proportion of contribution of each participant group to each analytical theme.

Our visualisation of the proportion of each theme which drew on findings from each of the three participant groups (Figure 2) shows that findings from patient participants contributed most to Themes 1 and 2. Theme 4 drew most on findings from healthcare professional participants, Theme 3 was roughly equal between patient and HCP participants. Findings from informal carer participants contributed the least overall, and, other than Theme 1, least to most themes.

#### Overwhelming situation of ‘no choice’

Our metasynthesis concluded that people’s experience and process of decision-making is strongly influenced by the serious implications of receiving a non-curative cancer diagnosis. Patients face decisions in a stressful situation where they are emotionally overwhelmed, and their abilities to receive information and be involved in decision-making are affected by strong emotions, a feeling of urgency and fear of decisional regret.^37–43^ These feelings can influence patients’ wish for and ability to participate in shared decision-making, as Lape et al.^40^ comment (p. 910):

*This diagnostic context* [patients with spinal metastatic disease], *including illness uncertainty, shaped participant preferences for how much agency to take in their own decision process and sometimes limited participants from fully engaging in the decision through independent research and critical conversations with their health-care providers.*

Patients, informal carers and healthcare professionals all often shared the perception of treatment decision-making in advanced cancer as a ‘no choice’-situation.^38–41,43–51^ In many studies, patient and informal carer participants remarked that the only available option was following the treatment plan offered by the physician.^38,39,46,47,50^ Participants seldom perceived no treatment as equivalent to treatment, and any choice between broadly equivalent options was perceived as limited, as an oncologist in De Snoo-Trimp et al.^44^ commented (p. 1184):

*The concept of “no best option” is, in my opinion, somewhat theoretical because, in practice, there is often a best option based on the tumour and the status of patients.*

Physician participants in some studies remarked that following treatment protocols was essential, and awareness of equipoise irrelevant.^37,44,52^ Further, even if physicians presented options, people with cancer and informal carers did not always perceive the presence of choice in the same way.^45,49^ This could be a result of reduced abilities to receive information in an overwhelming situation, but also because they might not be ready to consider alternatives to continuing treatment.

Our metasynthesis illustrates the mutual imperative to treat and fight against cancer, shared amongst all study participants, with both patients and physicians perceiving stopping treatment as ‘giving up’.^37,39,43,44,47,48,53,54^ Physicians’ perceptions of patients’ wishes for treatment might also make it difficult to suggest no treatment, and physicians could provide treatment plans without addressing existing uncertainty and treatment limitations, sometimes motivated by a wish to meet the patients’ expectations for treatment and to preserve hope.^37,41,43,47,55^

In line with the limited perception of choices, some studies showed limited discussions of pros and cons of different options.^37,40,41,44,55,56^ Medical specialists provided information on the suggested option, and at the same time tried to adapt the information to patients’ individual needs.^42,44^ For example, a doctor in McCullough et al.^43^ commented (p. 486):

*. . . information delivery from my point of view (is) so that I can get them to see from my perspective what the decisions are and give them enough information so that they feel part of that decision and – and then able to make that decision. And then answering any questions . . . and then giving them a time interval, which is appropriate for them.*

Health care professionals and patients emphasise giving and receiving information about suggested treatments. Both groups can perceive the provision of comprehensive information as involvement in decision-making,^43,48^ so perception of involvement does not necessarily include deciding between choices. However, preferences for quantities and details of information vary,^38–40,46,48^ so it can be challenging to provide suitable amounts of information.

Study participants with cancer indicate that balancing quality of life with the limited potential benefits from active treatment is important and may express doubt about the value of continuing treatment.^47^ Healthcare professionals also mention positive benefit-harm ratios as key factors in treatment decision-making.^43,49^ However, evidence underpinning decisions may be limited. Included studies found that participants might delay or avoid addressing or sharing deliberations on these issues.^43,54^ Another approach was to start a process of decision-making by adapting the treatment, as illustrated by the following quote by an oncologist in De Kort et al.^52^ (p.169):

*I already had my doubts, but he wanted it very much so we decided to evaluate his clinical improvement after one chemotherapy course, after which we would be able to decide again. Then his health deteriorated, but he was not yet ready for the idea of stopping the treatment. We then switched to an oral chemotherapy.*

#### Processes vary depending on the timings and nature of the decisions involved

Shared decision-making in the context of palliative care is a process over time, which does not always involve a single choice between two options.^38,39,52^ De Kort et al.^52^ clearly state (p. 168):

*The practice of palliative chemotherapy is much more complex than the choice with a dilemmic character, namely either palliative chemotherapy or no chemotherapy, that patients might experience. It is more about how much, how often, and how long.*

Care and treatment options may change, and people’s preferences for involvement, and the extent of that involvement, vary over time.^42,48,57,58^ Some of the included studies showed that being involved in early decision-making about palliative treatment could build a relationship for future end-of-life discussions, and, as situations cha-nged, physicians and nurses revisited previous information and engaged with people with cancer and their families to reflect on the current situation.^53,58–60^ Also, with limited expected benefit of treatment, more room was given to considerations of patients’ preferences, as De Snoo-Trimp et al.^44^ comment (p. 1184):

*Almost all medical specialists differentiated between first and subsequent lines of chemotherapy. Because the amount of expected benefit is limited in subsequent lines, physicians’ preference to start treatment was less strong, and they gave more room for the opinion of the patient.*

Our metasynthesis concludes that preferences for involvement and the degree experienced also vary depending on the issue where decision-making is required.^38,41,48,52,57^ It is important for patients to be involved in adapting treatment and in the decisions about their daily life and care,^38,41,48,52,57^ but as the next theme describes, treatment decision-making is often deferred to a trusted medical specialist.

#### The patient-physician dyad is central to decision-making, with surrounding support

All stakeholder perspectives included in this metasynthesis centralise medical specialists, who are regarded as the experts, responsible for making treatment decisions. Physicians perceive themselves as responsible,^37,42,44,48^ nurses experience physicians as having the leading role,^59,61^ and people with cancer and their informal carers rely on and trust physicians to be the expert and know what is in their patients’ best interests.^38–41,46,48–50,56,57^ Gregersen et al.^46^ (p.68) illustrate this:

*Most patients considered the physician to be the expert in the decision-making and relied on the physician’s competences. One patient said: It is difficult for me to choose because you don’t know what is best. The physician must know that. Similar to this, several patients expressed the same viewpoint with comments like: I don’t understand it. I am not a physician.*

Both people with cancer and physicians considered patie-nts less capable of making decisions, due to their lack of knowledge.^42,44,48,56^ Many patients therefore deferred decisions to physicians, and physicians also expressed that they already knew what patients preferred and that patients did not want responsibility in decision-making.^37,44,48^ Even when the medical specialist emphasised patient involvement and wanted the patient to make the final decision, it could be hard to achieve because the patient found it difficult.^37^ Some medical specialists also expressed lack of ex-perience, knowledge or confidence needed for shared decision-making.^43,47,54^

However, this shared perception of the medical specialist’s responsibility for decision-making did not lead to patients or their family not experiencing any involvement in treatment decision-making. Included studies showed examples of patients’ experiences of being involved at their preferred level, and that decisions could be a process of reaching a mutual agreement between the physician, patient and informal carers.^37,48,50,51,53,55,56^

While informal carers could be an important source of information, advice and emotional support for the patient, their role in shared decision-making was experienced as mainly participating in meetings with the medical specialist and supporting the patient.^38,39,50^ Some studies showed that difficulties could be experienced, like family members not being involved because patients wanted to protect them, or that healthcare professionals perceived family members influencing patients to continue treatment they might not want.^43,51^

The patient-physician dyad is central, and people with cancer, nurses and other healthcare professionals perceive that other health care professionals generally have limited roles in shared decision-making.^43,45,46,48,50,59–61^ However, other healthcare professionals facilitate and support shared decision-making in several ways.

Firstly, they may motivate and promote patient involvement, and create awareness of choice.^43,58,60,61^ As above, perceptions and awareness of equal choices can be limited, but one study^60,61^ found that nurses can enable this. Bos-van den Hoek et al.^61^ (p.302) states:

*Nurses reported that they may create choice awareness or inform patients about treatment options and the benefits or disadvantages of such treatments: “I think it’s important to discuss with patients that they can choose to start chemotherapy and that they can always reconsider their decision when they notice that the chemotherapy leads to many complaints and a terrible decline in their quality of life”.*

Secondly, other healthcare professionals also support shared decision-making by providing information to the medical specialist and advocating for patients, clarifying information for the patient, helping to clarify patients’ preferences and values, and checking the quality of decisions.^38,41,43,48,58–61^ Nurses can contextualise their decisional support because of their broad knowledge of patients, and they provide emotional support.^43,46,59,61^ As stated by one nurse:

*(. . .) often patients just want somebody just to listen to them so that they can talk (through) all the things going on around in their heads; and often just being able to verbalize that then they can come to their decision.* (Nurse, p. 24) ^59^

#### Level of involvement depends on interactions between individuals and systems

People with cancer and also health care professionals consider personal attributes such as experience, knowledge and communication skills as important for shared decision-making in palliative cancer care.^47,56,59–61^ Person-centred communication skills and the development of a trusting relationship are considered necessary to enable shared decision-making,^38,41,46,50,55,56,60–62^ as Lee et al.^62^ state (p.447):

*When patients felt that the health professional was someone that they could trust, they were able to be involved in decisions. In trusting the health care professional, patients would have confidence that the health professional could be relied upon to give good advice, make good decisions and to care for them.*

Perhaps paradoxically, a trusting relationship was also described as the basis for deferring decisions to trusted physicians.^38,56,57^ People with cancer and informal carers indicate that showing kindness and empathy are important personal qualities of healthcare professionals that influence building trust.^50,62^ Yet, while patients and informal carers appreciated emotional involvement, some physicians in the included studies described emotional involvement as interfering with their need for objective and evidence-based decision-making.^43,54^

In addition to individual factors like personal knowledge and experience, healthcare professionals’ abilities to involve people with cancer in shared decision-making are affected by organisations, and their structures and cultures.^47,59–62^ Included studies found that patients and healthcare professionals experience time and continuity of care as important for shared decision-making.^43,47,50,59–62^ The roles of nurses in particular were affected by physicians’ attitudes and behaviours, and the existence of a supportive culture towards involvement was found to be important.^59,61^ Supporting shared decision-making requires that the clinical team shares an understanding of treatment goals.^42,59–61^ For instance, Barthow et al.^59^ comment that access to information affects nurses’ contributions to decision-making (pp. 24, 26):

(. . .) *lack of clarity about treatment goals caused nurses to feel reticent to provide information or discuss treatment decisions.* (. . .) *Access to up-to-date and detailed verbal or written information varied according (to) the setting and role in which the nurse practiced and consequently impacted on their ability to engage in decision support.*

Chen et al.^56^ also found that the extent of teamwork and collaboration between the physician and other team members influenced patients’ experience of involvement in decision-making.

## Discussion

Shared decision-making is currently a focus of research across health care. Our review focussed specifically on shared decision-making in palliative cancer care, aiming to identify and synthesise qualitative data on how people with cancer, their informal carers and healthcare professionals experience and perceive shared decision-making in palliative cancer care in particular.

### Main finding

Our main finding is that shared decision-making in palliative cancer care is a complex process of many decisions, in a challenging, multifaceted and evolving situation with limited equipoise and choice. Involvement in shared decision-making in this context depends on both individual and system level factors, including the ability and opportunity to build trusting relationships between health care providers, people with cancer and informal carers.

### What this study adds, and implications for practice

Our metasynthesis contributes to discussions in this field in three key areas, which are all important for practice: the complexity of understanding shared decision-making, palliative care professionals’ personal and professional competencies and multidisciplinary relationships.

#### The complexity of understanding shared decision-making

Our metasynthesis illustrates an important perception for the palliative context: the feeling of having no options, or, from the professionals’ perspective, lack of equipoise. Professional equipoise is described as present in situations where evidence gives the clinician no clear preference, and shared decision-making is considered most applicable to these situations.^63^ Our metasynthesis also indicates that mutual perceptions that there are no suitable options result in limited discussions and deliberations concerning harms and benefits. However, there are commonly situations in palliative care where the evidence varies, or important trade-offs, such as side effects, need to be considered.^24^ If it is reasonable to compare options, these situations would also qualify as having clinical equipoise, making shared decision-making a preferable approach.^24^ Nevertheless, our metasynthesis shows that clinicians can perceive shared decision-making as less relevant when treatment could be offered according to guidelines, even in situations with uncertainty and limited evidence.^44,52^

Our metasynthesis highlights a fundamental challenge experienced in palliative treatment and care decisions: balancing uncertain benefit of treatments against their possible negative effects on quality of life. Physicians’ hesitance to address existing uncertainty and treatment limitations can be motivated by a wish to meet patients’ expectations for treatment and preserve hope for a cure. It is important to recognise that the responsibility to facilitate shared decision-making lies with the clinician, and our metasynthesis also shows that patients often choose to defer decisions to physicians, placing their trust in their expert knowledge. In situations of life limiting illness and existential uncertainty, clarifying patients’ preferences for involvement is necessary, because misguided attempts to involve patients in decision-making may cause them harm and distress.^24^ Consequently, shared decision-making requires a mutual wish and ability to share uncertainty and limits.

Our study connects to ongoing clinical and theoretical discussions of definitions of shared decision-making, how to understand it and when it is appropriate.^64^ Conceptual confusions about shared decision-making have been shown to undermine its implementation by multi-disciplinary healthcare professionals, and involvement of people at the end of life and their families is affected by professionals’ uncertainty about just how to share those decisions.^65^

Three of the studies included in our review concluded that shared decision-making was not achieved, when comparing the outcomes to their chosen definition of shared decision-making.^37–39^ Another study questioned whether shared decision-making is the right approach in situations where patients have recently received a diagnosis of advanced cancer.^48^ As our metasynthesis indicates, treatment decisions are not solely a preference-sensitive choice between equal options but can be complex and even existential. Furthermore, preferences for involvement in shared decision-making vary depending on the timing and the nature of the decisions involved. The misconception that shared decision-making is only relevant in situations of clinical equipoise therefore needs to be addressed. Hargraves et al.^66^ state that shared decision-making can be understood as a range of methods that vary substantially with different situations and purpose.^66^ In line with such an understanding, clinicians need to identify and correctly understand the character of the situation, know the decisional role preferences of people with cancer and their families, and adapt the approach to shared decision-making to the person and situation.

Our findings underline the importance of taking the context of a serious, life-limiting diagnosis into account, because this strongly influences the wish for and ability to be involved and the possibilities of identifying elements of choice. Strategies for implementing shared decision-making must therefore be broadened and need to include how clinicians communicate with people with cancer and their families, to make well-informed decisions about further actions in various situations, not solely about treatment or choices between equal options.

#### Personal and interpersonal capacities and possibilities in the interprofessional team

Shared decision-making in palliative cancer care is a complex process, and our metasynthesis also shows that involvement in shared decision-making depends on personal and interpersonal compe-tencies in the health professional team. Personal communication skills and the development of trusting relationships are important, in addition to organisational aspects like a supportive culture, for shared decision-making and interprofessional teamwork.^38,41–43,46–48,50,55,56,59–62^ The review by Kuosmanen et al.,^29^ similarly found that pre-requisites for participating in shared decision-making are interdisciplinary teamwork, open communication, good patient–healthcare professional relationships, a favourable environment and mutual exchange of information between patients and healthcare professionals.^29^

Most of the included studies in our metasynthesis addressed treatment decision-making and showed that the patient and the medical specialist are in the centre of decision-making for these decisions. Légaré et al.^67^ developed an interprofessional model of shared decision-making, making explicit the role of ‘decision coach’, which any member of the healthcare team can assume.^67^ Our review also identifies the supporting and facilitating role of other members of the interprofessional team, showing that, for instance, nurses contribute to different key elements of shared decision-making.^38,41,43,48,58,59,61^ However, several of these studies build on an understanding of shared decision-making as a step-by-step approach in equipoise situations,^59–61^ like the four step model by Stiggelbout et al.^3^ Such models may not necessarily be the most appropriate way to address the particular processes which arise in palliative care. They might however be developed and adapted to the palliative care context, including how members of the interprofessional team can complement each other.

#### Implications for future research

Our review identified gaps in knowledge that further research might address. Most included studies focussed on treatment decisions. Future studies could address other subjects requiring decisions, such as place-of-care or symptom management. Furthermore, given the understanding of shared decision-making as a process of multiple decisions, longitudinal designs could complement existing research, exploring how decisions evolve and change over time. Future res-earch might also explore a wider approach to shared decision-making, which might increase the relevance and fit to palliative and end-of-life situations in diverse cultural and geographical healthcare settings. Few studies explored the roles of informal carers, and evidence for their role in shared decision-making is still limited.

### Strengths and limitations

A strength of our review and metasynthesis is its integration of the perspectives of all potential participants in shared decision-making, not only people with palliative cancer, but also interprofessional healthcare teams and informal carers. The qualitative metasynthesis design, focussing specifically on palliative cancer care, enables this integration of qualitative findings and so the creation of new, deeper knowledge.

Limitations to our study resulted from the variety of papers included. Including only studies published in English or Scandinavian languages meant that we excluded studies from countries and areas publishing in other languages, which limits geographical and cultural diversity. All included studies were conducted in Western/Northern cultural contexts, and so our review presents a particular perspective on shared decision-making, with a particular cultural bias. For example, a broader cultural context would probably have broader perceptions of how families might be part of decision-making, because family have a more prominent role in other cultures.^68^

Decisions on inclusion and exclusion involved some subjective judgements, so double-blinded screening and ongoing discussions in the research team, were important for reliability and validity.

## Conclusion

Our metasynthesis shows that shared decision-making in palliative cancer care is complex, which affirms the importance of adapting shared decision-making to suit people living with life-limiting diagnoses. Shared decision-making does not simply involve choices between equal options, which seldom occur in decisions in palliative cancer care. Awareness of choices in situations of uncertainty needs to be addressed, and not limited to situations of professional equipoise.

In addition to the personal competencies which each clinician needs to engage in shared decision-making, the interprofessional team must complement each other in order to fully involve people with palliative cancer and their informal carers to the extent they prefer. A better shared understanding of the concept of shared decision-making is necessary, including consideration of whether implementing step-by-step approaches to shared decision-making is the best way to improve these complicated processes in palliative care. Future research might usefully explore whether an interprofessional model of shared decision-making can be adapted to the complexities of multiple decisions in palliative cancer care.

## Supplemental Material

sj-pdf-1-pmj-10.1177_02692163241238384 – Supplemental material for Shared decision-making in palliative cancer care: A systematic review and metasynthesis Supplemental material, sj-pdf-1-pmj-10.1177_02692163241238384 for Shared decision-making in palliative cancer care: A systematic review and metasynthesis by Jannicke Rabben, Bella Vivat, Mariann Fossum and Gudrun Elin Rohde in Palliative Medicine

sj-pdf-2-pmj-10.1177_02692163241238384 – Supplemental material for Shared decision-making in palliative cancer care: A systematic review and metasynthesis Supplemental material, sj-pdf-2-pmj-10.1177_02692163241238384 for Shared decision-making in palliative cancer care: A systematic review and metasynthesis by Jannicke Rabben, Bella Vivat, Mariann Fossum and Gudrun Elin Rohde in Palliative Medicine
