# Supplementary material for: Shared decision-making in palliative cancer care: A systematic review and metasynthesis
Source: Palliat Med. 2024 Mar 13;38(4):406–22. doi: 10.1177/02692163241238384 (PMC11025308; doi:10.1177/02692163241238384)
Supplement: sj-pdf-1-pmj-10.1177_02692163241238384 – Supplemental material for Shared decision-making in palliative cancer care: A systematic review and metasynthesis [file sj-pdf-1-pmj-10.1177_02692163241238384.pdf]

# Supplementary file A: Key terms and search strategies

## Key terms

| | |
|---|---|
| **Population/Context**[a] Keywords for palliative | Palliative, terminal, «end of life», «end-stage», death, dying, incurable, non/not curable, hospice, advanced/serious/sever (cancer) |
| AND | |
| **Population/Context** Keywords for cancer | Neoplasm, cancer, tumor/tumour, oncolog |
| AND | |
| **Phenomenon of interest** Keywords for shared decision-making | Decision making, Shared/Group/Family/Patient decision making Shared/sharing/informed/participat/involv/collaborat/cooperat/ support/process/aid decision/choice «Advance care planning» [b], «advance directive»[b] |
| AND | |
| **Study design** Keywords for qualitative studies | Qualitative studies, research, method, Phenomenology/ phenomenological, Hermeneutics/ hermeneutical, Ethnography /ethnographic Grounded theory, Interview, focus group Narrative, content, thematic analysis, experience, themes |

a) Because both patient, relatives and all HCPs were included, no search terms necessary (include all)

b) was included in search as an overlapping phenomenon that may also include findings about SDM, -delimited from inclusion in screening process

## Search Strategy Ovid (Embase, MEDLINE and APA PsycInfo)

**Database:**
Embase <1974 to 2023 Week 21>Ovid MEDLINE(R) ALL <1946 to May 31, 2023>APA PsycInfo <1806 to May Week 4 2023>

| # | Query |
|---|---|
| 1 | ((shared or sharing or informed or participat* or involv* or collaborat* or cooperat* or support* or process or aid) adj3 (decision* or choice)).ti,ab,id,kf. |
| 2 | Decision Making, Shared/ or shared decision making/ or Group Decision Making/ or Decision Making/ or choice behavior/ |
| 3 | ("Advance Care Planning" or "Advance Directive*").ti,ab,kf,hw,id. |
| 4 | ((decision* adj3 (making or treatment or care)).ti,ab,id,kf. or (choice adj3 (behavi* or treatment or care)).ti,ab,kf,id.) adj7 (patient* or family or families or relatives or "next of kin*" or caregiver*).ti,ab,kf,id. |
| 5 | or/1-4 |
| 6 | (Palliative or terminal* or "end of life" or "end-stage" or death or dying or incurable or hospice).ti,ab,hw,kf,id. |
| 7 | (("not" or non) adj2 (curabl* or curativ*)).ti,ab,kf,id. |
| 8 | ((Advanced or serious* or sever*) adj5 cancer).ti,ab,kf,id. |
| 9 | or/6-8 |
| 10 | exp Neoplasms/ or exp neoplasm/ |
| 11 | (cancer or tumor* or tumour or neoplasm* or oncolog*).ti,ab,kf,id,hw. |
| 12 | 10 or 11 |
| 13 | (Qualitative Study or interview).md. |
| 14 | exp qualitative methods/ or exp qualitative research/ or Phenomenology/ or ethnography/ or Hermeneutics/ or narrative analysis/ or content analysis/ or Focus Groups/ or Hermeneutics/ or Grounded Theory/ or qualitative analysis/ or thematic analysis/ |
| 15 | (Qualitative or Interview* or phenomenolog* or Experience* or themes or thematic or ethnograph* or "Focus Group*" or "grounded theor*" or hermeneutic* or "content analys*").ti,ab,hw,kf,id. |
| 16 | or/13-15 |
| 17 | 5 and 9 and 12 and 16 |
| 18 | Conference Abstract.pt. |
| 19 | 17 not 18 |
| 20 | remove duplicates from 19 |

## Advanced Search in Scopus

**Shared …**
( TITLE-ABS-KEY ( ( shared OR sharing OR informed OR participat* OR involv* OR collaborat* OR cooperat* OR support* OR process OR aid ) W/2 ( decision* OR choice ) ) OR TITLE-ABS-KEY ( "Advance Care Planning" OR "Advance Directive*" ) OR TITLE-ABS-KEY ( ( ( decision* W/2 ( making OR treatment OR care ) ) OR ( choice W/2 ( behavi* OR treatment OR care ) ) ) W/6 ( patient* OR family OR families OR relatives OR "next of kin*" OR caregiver* ) ) )

**AND Palliative…**
( TITLE-ABS-KEY ( palliative OR terminal* OR "end of life" OR "end-stage" OR death OR dying OR incurable OR hospice ) OR TITLE-ABS-KEY ( ( "not" OR non ) W/1 ( curabl* OR curativ* ) ) OR TITLE-ABS-KEY ( ( advanced OR serious* OR sever* ) W/4 cancer ) )

**AND Cancer…**
TITLE-ABS-KEY ( cancer OR tumor* OR tumour OR neoplasm* OR oncolog* )

**AND qualitative studies…**
TITLE-ABS-KEY ( qualitative OR interview* OR phenomenolog* OR experience* OR themes OR thematic OR ethnograph* OR "Focus Group*" OR "grounded theor*" OR hermeneutic* OR "content analys*" )

**Combined – advanced search:**
( ( TITLE-ABS-KEY ( ( *shared* OR *sharing* OR *informed* OR *participat** OR *involv** OR *collaborat** OR *cooperat** OR *support** OR *process* OR *aid* ) W/2 ( *decision** OR *choice* ) ) OR TITLE-ABS-KEY ( *"Advance Care Planning"* OR *"Advance Directive*"* ) OR TITLE-ABS-KEY ( ( ( *decision** W/2 ( *making* OR *treatment* OR *care* ) ) OR ( *choice* W/2 ( *behavi** OR *treatment* OR *care* ) ) ) W/6 ( *patient** OR *family* OR *families* OR *relatives* OR *"next of kin*"* OR *caregiver** ) ) ) ) AND ( ( TITLE-ABS-KEY ( *palliative* OR *terminal** OR *"end of life"* OR *"end-stage"* OR *death* OR *dying* OR *incurable* OR *hospice* ) OR TITLE-ABS-KEY ( ( *"not"* OR *non* ) W/1 ( *curabl** OR *curativ** ) ) OR TITLE-ABS-KEY ( ( *advanced* OR *serious** OR *sever** ) W/4 *cancer* ) ) ) AND ( TITLE-ABS-KEY ( *cancer* OR *tumor** OR *tumour* OR *neoplasm** OR *oncolog** ) ) AND ( TITLE-ABS-KEY ( *qualitative* OR *interview** OR *phenomenolog** OR *experience** OR *themes* OR *thematic* OR *ethnograph** OR *"Focus Group*"* OR *"grounded theor*"* OR *hermeneutic** OR *"content analys*"* ) )

## CINAHL (EBSCO*host*). Search modes - Boolean/Phrase

Note: The default fields for unqualified searches consist of the following: Title, Abstract and Subject headings, PubMed ID (PMID), Digital Object Identifier, Author.

| # | **Query** |
|---|---|
| S1 | (shared or sharing or informed or participat* or involv* or collaborat* or cooperat* or support* or process or aid) N2 (decision* or choice) |
| S2 | MH "Decision Making" OR MH "Decision Making, Family" OR MH "Decision Making, Patient" OR MH "Decision Making, Shared" |
| S3 | "Advance Care Planning" OR "Advance Directive*" |
| S4 | ((decision* N2 (making or treatment or care)) or (choice N2 (behavi* or treatment or care))) N6 (patient* or family or families or relatives or "next of kin*" or caregiver*) |
| S5 | S1 OR S2 OR S3 OR S4 |
| S6 | Palliative or terminal* or "end of life" or "end-stage" or death or dying or incurable or hospice |
| S7 | ("not" or non) N1 (curabl* or curativ*) |
| S8 | (Advanced or serious* or sever*) N4 cancer |
| S9 | S6 OR S7 OR S8 |
| S10 | (MH "Neoplasms+") |
| S11 | cancer or tumor* or tumour* or neoplasm* or oncolog* |
| S12 | S10 OR S11 |
| S13 | (MH "Qualitative Studies+") |
| S14 | Qualitative or Interview* or phenomenolog* or Experience* or themes or thematic or ethnograph* or "Focus Group*" or "grounded theor*" or hermeneutic* or "content analys*" |
| S15 | S13 OR S14 |
| S16 | S5 AND S9 AND S12 AND S15 |
