# Supplementary material for: Shared decision-making in palliative cancer care: A systematic review and metasynthesis
Source: Palliat Med. 2024 Mar 13;38(4):406–22. doi: 10.1177/02692163241238384 (PMC11025308; doi:10.1177/02692163241238384)
Supplement: sj-pdf-2-pmj-10.1177_02692163241238384 – Supplemental material for Shared decision-making in palliative cancer care: A systematic review and metasynthesis [file sj-pdf-2-pmj-10.1177_02692163241238384.pdf]

Supplementary file B Study characteristics, additional

Author (Year), Geographic location of study	Title	Data collection	Sampling Method	Cancer Diagnosis	Study setting	Theoretical framework	References to definition /models of SDM	Purpose/Aim
Al Achkar et al., (2022), United States	A qualitative study of interactions with oncologists among patients with advanced lung cancer.	Semi-structured interviews (in-person, telephone, or videoconference)	Purposive	Lung cancer	Unclear; receiving medical care/recruited from online support groups	RM DER (2012) Self-determination theory	-	To (1) understand the perspectives of advanced lung cancer patients with oncogenic alterations about their care experiences with their oncologist(s), and (2) identify the perceptions of advanced lung cancer patients on seeking second opinions and navigating care decisions.
Barthow et al., (2009), New Zealand	To be involved or not: factors that influence nurses' involvement in providing treatment decisional support in advanced cancer	Face-to-face, in-depth semi-structured interviews	Purposive (as stated in McCullough)	Variable	Tertiary cancer centre including in-patient, ambulatory, community, and palliative care	-	Brundage et al., (2005); Elwyn et al., (1999); Gattelari et al., (2001)	To elucidate and describe the nature of factors that impact on nurses' involvement in decisional support
Beaussant et al., (2015), France	Is shared decision-making vanishing at the end-of-life? A descriptive and qualitative study of advanced cancer patient's involvement in specific therapies decision-making	Questionnaire and in-depth interviews using semi-directive topic lists	Convenience (consecutive)	Variable	Hospital/ cancer centre	-	-	To explore (1) the frequency of the questioning about the pursuit, the limitation, or the withholding of ST in advanced cancer patients, and (2) the determinants and modalities of these questionings from the compared physicians' and the patients' perspectives, with a focus on the patients' involvement.
Boele et al., (2023), United Kingdom	Communication in the context of glioblastoma treatment: A qualitative study of what matters most to patients, caregivers and health care professionals.	Semi-structured interviews by telephone or videoconference	Convenience and purposive	Glioblastoma	Hospital	-	-	To explore experiences and preferences around glioblastoma treatment communication in patients, family caregivers and healthcare professionals.
Bos-van den Hoek (2021), Netherlands	The role of hospital nurses in shared decision-making about life-prolonging treatment: A qualitative interview study	Face-to-face, in-depth, semi-structured interviews	Purposive	Variable	Hospital; in-patient, out-patient and day care		Charles et al., (1997); Elwyn et al., (2017); Stiggelbout et al., (2015)	To examine hospital nurses' perception of their actual and potential contribution to shared decision-making about life-prolonging treatment and their perception of the pre-conditions for such a contribution.
Bos-van den Hoek (2022), Netherlands	Role of GPs in shared decision making with patients about palliative cancer treatment: a qualitative study in the Netherlands	Face-to-face, in-depth, semi-structured interviews	Purposive and convenience	Variable	General practice, (hospital treatment)		Legare & Witteman (2013), Charles et al., (1997); Stiggelbout et al., (2015)	To explore GPs' perspectives on their role in SDM about palliative cancer treatment and the requirements they report to fulfil this role.
Brom et al., (2017), Netherlands	Challenges in shared decision making in advanced cancer care: a qualitative longitudinal observational and interview study	Face-to-face interviews with a topic list and observations of clinic visits	Convenience (consecutive)	Glioblastoma, colorectal cancer	Hospital; out-patient	Stiggelbout et al., (2012) Four step-model	Additional references: Charles et al., (1997, 1999); Makoul & Clayman, (2006); Towle & Godolpin, (1999)	To gain insight into treatment decision making in the last phase of life of advanced cancer patients by examining whether the steps of SDM are applied in clinical practice.
Chen et al., (2021), United States	Patient-Provider Communication, Decision-Making, and Psychosocial Burdens in Palliative Radiotherapy: A Qualitative Study on Patients' Perspectives	Oral questionnaires and Semi-structured interviews	Convenience (consecutive)	Variable	Comprehensive cancer centre	-	-	To examine patient perceptions of discussions, decision-making, and psychosocial burdens related to receiving palliative radiotherapy (RT), in order to inform best practices for communication about palliative RT.
De Kort et al., (2010), Netherlands	Understanding palliative cancer chemotherapy: about shared decisions and shared trajectories	Longitudinal with repeated semi-structured interviews, single interviews, focus groups and observations	Purposive	Colorectal cancer, pancreatic cancer	Hospital; out-patient	-	Charles et al., (1997); Emanuel & Emanuel, (1992)	To analyse these processes (decision-making during the treatment course of metastatic cancer patients) as trajectories.

Author (Year), Geographic location of study	Title	Data collection	Sampling Method	Cancer Diagnosis	Study setting	Theoretical framework	References to definition /models of SDM	Purpose/Aim
De Snoo-Trimp et al., (2015), Netherlands	Perspectives of Medical Specialists on Sharing Decisions in Cancer Care: A Qualitative Study Concerning Chemotherapy Decisions With Patients With Recurrent Glioblastoma	Interviews and focus group with topic lists	Convenience (consecutive) and purposive	Glioblastoma	Hospital	Stiggelbout et al., (2012) Four step-model	Additional references: Brock, (1991); Charles et al., (1997)	To describe the perspectives of medical specialists on the decision-making process with patients with glioblastoma
Elit et al., (2003), Canada	Women's perceptions about treatment decision making for ovarian cancer	Semi-structured interviews	Convenience	Ovarian cancer	Hospital	-	Charles et al., (1997)	To explore patients' perspectives on treatment decision making with their doctors, in particular, their experiences, perceptions, and constructions of meaning.
Elit et al., (2010), Canada	It's a choice to move forward: Women's perceptions about treatment decision making in recurrent ovarian cancer	Semi-structured interviews	Convenience	Ovarian cancer	Cancer centre; out-patient	-	Charles et al., (1997, 1999)	To explore women's perspectives about their TDM process.
Gregersen et al., (2022), Denmark	Patients' experiences of the decision-making process for clinical trial participation	Semi-structured, in-depth interviews	Convenience	Variable	Hospital, out-patient	-	-	To explore patients' experiences of the decision-making process when patients are presented with the opportunity to participate in a cancer clinical trial and to shed light on how patients experience the health communication, the nurse's role, and the physician's role.
Haun et al., (2022), Germany	Negotiating decisions on aggressive cancer care at end-of-life between patients, family members, and physicians - A qualitative interview study	Semi-structured interviews	Purposive	Variable	Cancer centre	Herbert A. Simon theory on decision-making	-	To give a thorough understanding of the involvement of family members of advanced-stage cancer patients (in the decision-making process)
Kvåle & Bondevik, (2008), Norway	What is important for patient centred care? A qualitative study about the perceptions of patients with cancer	In-depth interviews	Purposive	Variable	Hospital; in-patient	-	Frosh &Kaplan (1999)	To get insight in patients with cancers' perceptions of the importance of being respected as partners and share control of decisions about interventions and management of their health problems and the reasons behind their wishes.
Lape et al., (2020), United States	Patient experiences of decision-making in the treatment of spinal metastases: a qualitative study	Semi-structured interviews (primarily by phone)	Convenience	Variable	Tertiary medical centres	-	-	To understand factors that influenced decision-making around care for spinal metastases.
LeBlanc et al., (2018), United States	Triadic treatment decision-making in advanced cancer: a pilot study of the roles and perceptions of patients, caregivers, and oncologists	Survey and semi-structured interviews	Convenience and purposive	Gastrointestinal cancer or hematologic malignancy	Cancer centre; out-patient	-	Joseph-Williams et al., (2014), Elwyn et al., (2012)	To explore how all members of the triad (patients, oncologists, and caregivers) perceive and experience decisions related to treatment for advanced cancer.
Lee et al., (2009), Australia	Professional relationships in palliative care decision making	Interviews and field observations	Purposive	Variable[1]	Hospital, hospice, and community	-	-	To describe the significant issues that influence the processes of care decision making from the perspective of patients with advanced illness.
Løwe et al., (2021), Denmark	The Balance of Patient Involvement: Patients' and Health Professionals' Perspectives on Decision-Making in the Treatment of Advanced Prostate Cancer	Longitudinal with participant observation and repeated interviews	Purposive	Prostate cancer	Hospital; out-patient	Annemarie Mol (2008) theoretical perspectives on the logic of care and the logic of choice	Ahmad et al., (2014), Pieterse at al., (2018), Stiggelbout et al., (2015)	To explore the attitude of patients with APC to involvement in treatment decisions and how physicians' and nurses' approaches to patient involvement were expressed through attitude and action. Furthermore, the aim was to explore whether decision-making changes as the treatment course progresses.
McCullough et al., (2010), New Zealand	A model of treatment decision making when patients have advanced cancer: how do cancer treatment doctors and nurses contribute to the process?	Face-to face, In-depth, semi-structured interviews	Purposive	Variable	Tertiary cancer centre including in-patient, ambulatory (out-patient), community and palliative care)	-	-	To describe from the perspective of doctors and nurses their contributions to the treatment-related decision-making process when patients have advanced cancer.
Norton et al., (2019), United States	Family caregiver descriptions of stopping chemotherapy and end-of-life transitions	Semi-structured interviews	Convenience (consecutive)	Variable	Unclear, part of larger multi-centre RCT-study	-	-	To describe family caregivers' perspectives of the final month of life of patients with advanced cancer, particularly whether and how chemotherapy was discontinued and the effect of clinical decision-making on family caregivers' perceptions of the patient's experience of care at the end of life (EOL).

[1] Two of included patients had non-cancer diagnosis.

Author (Year), Geographic location of study	Title	Data collection	Sampling Method	Cancer Diagnosis	Study setting	Theoretical framework	References to definition /models of SDM	Purpose/Aim
Pfeil et al., (2015), Germany	What Keeps Oncologists From Addressing Palliative Care Early on With Incurable Cancer Patients? An Active Stance Seems Key	In-depth, semi-structured interviews	Unclear	Variable	Hospital	-	-	To explore the factors that hinder patient involvement in decision-making, and to reconstruct how physicians and nurses in oncology perceive their roles in preparing patients for EOL decisions
Robijn et al., (2018), Belgium, United Kingdom and the Netherlands	The involvement of cancer patients in the four stages of decision-making preceding continuous sedation until death: A qualitative study	Semi-structured interview (using an aide mémoir)	Purposive	Variable	Hospital, palliative care unit, hospice, and community	Charles, Gafni, & Whelan (1999) model of SDM	Additional reference: Elwyn et al.,(2012)	To describe the decision-making process preceding continuous sedation until death with particular attention to the involvement of the person who is dying.
Sowerbutts et al., (2020), United Kingdom	Discharging Women with Advanced Ovarian Cancer on Home Parenteral Nutrition: Making and Implementing the Decision	Longitudinal, repeated in-depth interviews	Unclear	Ovarian cancer	Hospital, community	-	Charles et al., (1999)	To explore the decision-making process for HPN in patients with ovarian cancer in bowel obstruction and to investigate barriers and facilitators to implement the decision.
Tarberg et al., (2022), Norway	Physicians' perceptions of patient participation and the involvement of family caregivers in the palliative care pathway	In-depth, semi-structured interviews	Purposive	Variable	Hospital and primary care	Beuchamp & Childress' (2013) four principles of biomedical ethics Thompson et al. (2007) five levels of involvement	-	To explore physicians' perceptions of patients and family caregivers' involvement in the different phases of the palliative pathway
Van Oosterhout et al., (2021), Netherlands	Experiences of bereaved family caregivers with shared decision making in palliative cancer treatment: a qualitative interview study	In-depth interviews with the use of a topic list	Purposive	Variable	Hospital	Models of SDM by Elwyn et al.(2012, 2017), and van de Pol et al. (2019)	Additional references: Charles et al., (1997)	To explore how bereaved family caregivers experienced the shared decision-making process.
